# Mechanisims of asthma and allergic disease – 1080. Interactive effect of sodium sulfite and rhinovirus infection in chemokines production by airway epithelial cells

**DOI:** 10.1186/1939-4551-6-S1-P76

**Published:** 2013-04-23

**Authors:** Hyun Hee Kim, Woo-Jin Chung, Yoon Hong Chun, Jong-Seo Yoon, Joon Sung Lee

**Affiliations:** 1 Pediatrics, Bucheon St. Mary's Hospital, College of Medicine the Catholic University of Korea, Seoul, South Korea; 2 Seoul St. Mary's Hospital College of Medicine the Catholic University of Korea, South Korea; 3 Pediatrics, Incheon St. Mary's Hospital, South Korea

## Background

Sodium sulfite (Na_2_SO_3_) is a product of sulfur dioxide (SO_2_). Inhaled sulfur dioxide can be easily hydrated to yield sodium sulfite in the respiratory tract. Sulfur dioxide is one of the most important air pollutants that can adversely affect the respiratory system. Rhinovirus (RV) is a major cause of common cold and is a major risk factor responsible for the exacerbation of asthma and chronic obstructive pulmonary disease. An epidemiological study suggested that interactions between sulfur dioxide and viral infections exacerbate respiratory disease. However, little is known about the mechanism underlying these interactions. We investigated the effects of sodium sulfite on the production of RV-induced chemokines such as interleukin-8 (IL-8); regulated on activation, normal T-cell expressed and secreted (RANTES); and interferon-gamma inducible protein-10 (IP-10) in airway epithelial cells in vitro.

## Methods

A549 airway epithelial cells were pretreated with 2,500 μM sodium sulfite for 6 h at 37°C and infected with RV-7 at 1 × 10©ù tissue culture infectious dose 50% (TCID50)/mL for 2 h at 33°C. The medium was replaced with a virus-free medium, and the cells were incubated for 40 h at 33°C. Cell culture supernatants and mRNA were harvested at 24 h and 48 h after sodium sulfite treatment. Production and mRNA expression of IL-8, RANTES, and IP-10 in these harvests were assessed by ELISA and real-time PCR.

## Results

RV induced the production and mRNA expression of IL-8, RANTES, and IP-10 in the A549 cells. Sodium sulfite did not affect the viability of A549 cells or RV replication under our experimental conditions. When the cells were pretreated with sodium sulfite prior to RV infection, production and mRNA expression of RV-induced IL-8, RANTES, and IP-10 were enhanced with no effect on cell viability or RV replication.

## Conclusions

Our results suggest that sodium sulfite may potentiate the activity of RV-induced diseases by increasing the production of IL-8, RANTES, and IP-10.

